# The Role of the PFNA Operon of Bifidobacteria in the Recognition of Host’s Immune Signals: Prospects for the Use of the FN3 Protein in the Treatment of COVID-19

**DOI:** 10.3390/ijms22179219

**Published:** 2021-08-26

**Authors:** Venera Z. Nezametdinova, Roman A. Yunes, Marina S. Dukhinova, Maria G. Alekseeva, Valery N. Danilenko

**Affiliations:** 1 Laboratory of Bacterial Genetics, The Vavilov Institute of General Genetics, 117971 Moscow, Russia; veneranez@rambler.ru (V.Z.N.); romanyunes@gmail.com (R.A.Y.); alekseevamg@mail.ru (M.G.A.); 2 International Institute ‘Solution Chemistry of Advanced Materials and Technologies’, ITMO University, 197101 Saint-Petersburg, Russia; dukhinova@scamt-itmo.ru

**Keywords:** bifidobacteria, PFNA operon, host-microbe interaction, immune system, evolution

## Abstract

Bifidobacteria are some of the major agents that shaped the immune system of many members of the animal kingdom during their evolution. Over recent years, the question of concrete mechanisms underlying the immunomodulatory properties of bifidobacteria has been addressed in both animal and human studies. A possible candidate for this role has been discovered recently. The PFNA cluster, consisting of five core genes, *pkb2*, *fn3*, *aaa-atp*, *duf58*, *tgm*, has been found in all gut-dwelling autochthonous bifidobacterial species of humans. The sensory region of the species-specific serine-threonine protein kinase (PKB2), the transmembrane region of the microbial transglutaminase (TGM), and the type-III fibronectin domain-containing protein (FN3) encoded by the I gene imply that the PFNA cluster might be implicated in the interaction between bacteria and the host immune system. Moreover, the FN3 protein encoded by one of the genes making up the PFNA cluster, contains domains and motifs of cytokine receptors capable of selectively binding TNF-α. The PFNA cluster could play an important role for sensing signals of the immune system. Among the practical implications of this finding is the creation of anti-inflammatory drugs aimed at alleviating cytokine storms, one of the dire consequences resulting from SARS-CoV-2 infection.

## 1. Introduction

Today, viral infectious diseases continue to pose a palpable threat to healthcare around the world. Both old and recently evolved viruses, especially those capable of causing respiratory, intestinal and urogenital infections, fall into the category of dangerous infectious agents. The ongoing pandemic of SARS-CoV-2 exposed the tragic shortage of effective and affordable antiviral drugs, let alone anti-inflammatories capable of suppressing cytokine storms and restoring the balance between the innate and the adaptive immune systems. Unfortunately, despite the raging health crisis, the antiviral properties of probiotic bacteria such as bifidobacteria remain largely marginalized [1].

Bifidobacteria are a genus of one of the largest taxonomic units of the domain Bacteria, the phylum Actinobacteria [2]. Bifidobacteria are also some of the oldest bacteria that inhabited Earth long before the oxygenation of the atmosphere [3,4]. At the time of writing, the genus Bifidobacterium consisted of 86 species and 13 subspecies, which amounts in total to 99 taxa [5,6,7,8]. Bifidobacteria are the sole representatives of Actinobacteria that inhabit the gastroenterological tract (GIT) [2]. The ecological niches occupied by bifidobacteria are not limited to the intestines of mammals, birds, and social insects [8,9,10,11,12]. Bifidobacteria are also found in the human oral cavity and play a dual role there in relation to dental and gum health [13,14]. *B. dentium* is associated with caries and tooth decay [15]. Probiotic species of bifidobacteria have shown positive results in the treatment and prevention of gingivitis and periodontitis [16]. Bifidobacterial strains are usually adapted to their ecological niche, whether it is the intestines of a specific animal species or the human intestine [8,17,18]. How these different ecological specializations are underpinned genetically remains an open question. Evolutionarily, the gradual bifidobacterial colonization of anaerobic GITs of animals was driven by selective pressure from the host immune system. All the while, bifidobacteria effectively colonized newly encountered niches due to their ability to efficiently utilize the glycans of the mucus layer that overlies the intestinal epithelium [19,20,21]. One of the main challenges that bifidobacteria had to overcome when they started colonizing animal cavities was the circumvention of the host’s immune system. It can be assumed that the process of transformation of bifidobacteria into symbiotic bacteria occurred through co-evolution and mutual adaptation. It is not unlikely that bifidobacteria took an active part in the formation of the immune system of animals that they inhabited the same way they forge the immune system of newborn children. Co-evolution of bifidobacteria and their hosts possibly led to the formation of specialized mechanisms and operons in all members of the genus, ultimately allowing a sustained balance with the host’s immune system to take place. The discovery that FN3 can selectively bind to tumor necrosis factor TNF-α has opened many perspectives [22].

Research in this field was grounded in the hypothesis that the evolution of bifidobacteria, favoring those that better adapted to their hosts, must have led them to develop genes and species-specific mechanisms that increased their chances of colonization of new niches and efficient interaction with the host organism. This process might have occurred multiple times at different stages of the evolution of the immune system [23]. Many recent findings suggest that the symbiotic intestinal microbiota, and bifidobacteria in particular, not only participate in metabolic regulation but also shape the host immunity [24,25,26,27]. Bacteria constantly interact with immune and intestinal endothelial cells controlling the synthesis of various cytokines and thereby modulating the host’s immune response [28,29]. Bacteria are also capable of recognizing host-derived chemicals using membrane receptors that act as switches of signal transduction systems [30]. For example, bifidobacteria are capable of reducing the viral titer of acute respiratory tract infections via activation of the human immune system and modulation of cytokine production [31]. Thus, the potential of bifidobacteria and their metabolites for immune regulation can be put to use for the treatment of acute upper respiratory infections caused by viruses such as SARS-CoV-2 [32]. The immunomodulatory properties of bifidobacteria are already being used in the complex therapy of various diseases, including cancer [33], lung disease [34], and gastrointestinal diseases [35]. In recent years, a few reviews have scrutinized the role of bifidobacteria and probiotics containing them in modulating the host’s immune system [36,37,38]. This review discusses the bifidobacterial genes and their products involved in immune regulation in humans with a focus on the recently discovered PFNA operon. It also discusses the prospects for using the PFNA operon for inhibiting the “cytokine storm” caused by SARS-CoV-2 as an alternative for anti-TNF-α and -IL-6 monoclonal antibodies.

## 2. Bifidobacteria and the Human Immune System

Bifidobacteria are anaerobic Gram-positive polymorphic branched rods whose genomes are characterized by high GC content. The relative abundance of bifidobacteria in the human gut microbiome reaches its highest levels in naturally born and breastfed infants and gradually dwindles throughout a person’s childhood and adolescence to a few percent by adulthood [39,40]. Most importantly, bifidobacteria constitute the bulk probiotic species of the human gut microbiota. The gut microbiota of breastfed infants in the norm consists of the species *B. breve*, *B. bifidum*, *B. longum *subsp.* infantis* and *B. longum *subsp.* longum*, which are deemed typical residents of human infants [41]. A typical adult microbiota is marked by the presence of the species *B. adolescentis* and *B. catenulatum*, *B. pseudocatenulatum* and *B. longum *subsp.* longum* [42]. Some species such as *B. longum *subsp.* longum* were reported as the dominant bifidobacterial species in both infants and adults. *B. adolescentis* is the most common bifidobacterial species in adults. There is both genotypic and phenotypic evidence that *B. adolescentis* strains possess broad metabolic capabilities enabling them to utilize many dietary glycans such as starch, poly- and oligosaccharides, amylopectin, pullulan, maltotriose, and maltodextrin. Conversely, *B. adolescentis* strains lack the necessary genes for host glycan metabolism, such as mucin and breast milk oligosaccharides, which sets them apart from other infant-type bifidobacteria [43]. Although bifiddobacteria counts tend to be low in adults, *B. adolescentis* and *B. longum *subsp.* longum* are often prevalent in centenarians [27].

The gut microbiota serves as a major regulator of the development of the immune system of infants, which is further supported by the fact that late colonization of the baby’s intestines or acquisition of a microbiota relatively low in complexity can translate into slower maturation of the adaptive immune system. Moreover, bifidobacterial colonization of one-week-old newborns is associated with higher production of IL-5, IL-6, IL-13, TNF-α by the age of three years. In contrast, intestine colonization by Enterococcus spp., *Staphylococcus aureus* or *Clostridium* spp. in early infancy results in lower production of IL-13, IL-5 and TNF-α at three years of age. Early-life intestine colonization by bifidobacteria also affects T-cell maturation [44].

Interestingly, some bifidobacterial species such as *B. adolescentis*, *B. pseudocatenulatum*, and *B. longum* exhibit both pro-inflammatory and anti-inflammatory properties. For instance, studies have shown that the expression of the inflammatory cytokines TNF-α and IL-1 and the activity of natural killer cells are boosted by bifidobacteria, which holds great potential for treating immunocompromised patients. Contrastingly, some strains exhibit anti-inflammatory effects by reducing TNF-α, IL-8 and IL-1β levels [45,46]. Such strains could become effective drugs against inflammatory diseases exemplified by colitis and LPS-related conditions. In the case of *S. aureus* and *K. pneumoniae* infections, bifidobacteria are capable of immune system modulation via induction or suppression of both pro- and anti-inflammatory cytokines [37]. A number of *B. pseudocatenulatum* strains have been shown to suppress TNF-α and IL-6 production while stimulating IL-10 production [46]. Overall, these results corroborate the efficacy of Bifidobacterium strains as potential solutions for infectious and inflammatory diseases [37].

The gut microbiota as an ecosystem encapsulated in the human body has a great impact on health. As mentioned before, the importance of the gut microbiota springs from its involvement in immune system modulation. This function manifests itself via enhancement of the host’s defense mechanisms by maintaining a functional mucosal barrier and well-honed immune responses [47,48]. Bifidobacteria constitute a significant part of any healthy gut microbiome. They are crucial for the preservation of favorable intestinal environment and exert an immunoregulatory function as shown on animals and humans [27,48,49].

Bifidobacteria and the host’s immune system mutually interact with each other. There is mounting evidence that the immunomodulatory properties of bifidobacteria, which can be both species- and strain-specific, are often explained by their cellular components and metabolites [23,24,50,51]. Evolutionarily speaking, gut bacteria, whether commensal or pathogenic, experience constant pressure from the host immune system. As is the case of any ecosystem, the survival of a species depends primarily on its ability to efficiently react and adapt to changing environmental conditions. Thus, the adaptive potential of bacteria can depend to a great extent on the efficiency of their internal and external signaling pathways. During evolution, microorganisms have developed sensory systems allowing them to probe the health of their host by intercepting signals released by the immune system [24,52]. Unfortunately, these mechanisms remain poorly understood. Only a handful of studies, albeit carried out on pathogenic bacteria, demonstrated bacteria’s ability to bind to host cytokines [30,53]. The domains of bacterial proteins capable of interaction with host cytokines are in some cases structurally similar to other proteins involved in cytokine signaling. For instance, the IrmA protein of *E. coli*, which is structurally similar to interleukin receptors, is capable of binding to human cytokines IL-2, IL-4 and IL-10. The spatial structure of this protein revealed the presence of a fold similar to the structure of the human FN3 [30]. Today, the literature on receptors of commensal bacteria that specialize in the recognition of immune signals is scarce to nonexistent. The PFNA operon is one of the few known examples in bifidobateria [22,23].

The PFNA operon contains at least three genes (*pkb2*, *tgm* and *fn3*) potentially involved in the recognition of signals of the host immune system. PKB2 is a serine-threonine protein kinase containing a variable extracellular sensory region in different species of bifidobacteria. PKB2 phosphorylates some of the proteins encoded by the PFNA operon as well as a number of other extracellular moonlighting proteins [54]. The FN3 membrane protein contains two fibronectin domains including cytokine binding motifs, which allowed the strain *B. longum* GT15 to selectively bind in an in vitro setting TNF-α [22]. A fundamentally important characteristic of the PFNA operon is the species-specificity characterized by high divergence of some of the genes comprising it compared to the core genes of the operon. It has been shown that this divergence is likely the result of positive selection pressure [23,55,56]. Taken together, this prompted us analyze the available data on the structure of the known functions of the PFNA operon. The results of this analysis are presented in the following sections.

## 3. Species-Specific PFNA Operon, Structure, Possible Functions

**Figure 1:**
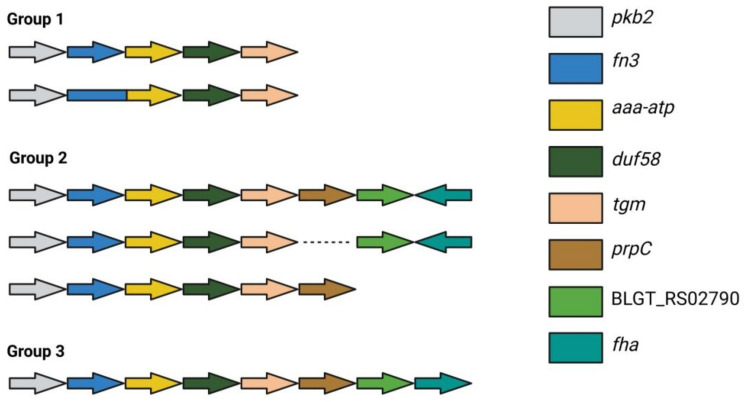
Structure of the PFNA operon across different members of the genus *Bifidobacterium*. Group 1—the cluster consists of five genes: *B. actinocoloniiforme*, *B. angulatum*, *B. asteroides*, *B. biavatii*, *B. coryneforme*, *B. indicum*, *B. lemurum*, *B. merycicum*, *B. scardovii*, *B. thermacidophilum* (group 1) and *B. breve* (there is fusion of genes encoding the fibronectin type III domain-containing protein and AAA-ATPase). Group 2—the cluster contains between six and seven genes: *B. adolescentis*, *B. animalis*, *B. bohemicum*, *B. bombi*, *B. boum*, *B. catenulatum*, *B. choerinum*, *B. commune*, *B. cuniculi*, *B. dentium*, *B. gallicum*, *B. moukalabense*, *B. pseudocatenulatum*, *B. pseudolongum*, *B. reuteri*, *B. ruminantium*, *B. thermophilum* and *B. kashiwanohense* (the gene prpC is absent), *B. stellenboschense* (the gene BLGT_RS02790 is absent). Group 3—the cluster contains eight genes: *B. aesculapii*, *B. bifidum*, *B. longum*, *B. saguini*. The arrows indicate the direction of transcription of the genes [54]. Adapted from [60].

The involvement of genes encoding signal transduction systems in the interaction with the host’s immune system served as a premise for a series of studies. First, the nucleotide sequences of the six genes encoding serine-threonine protein kinases (STPKs) were compared among different species of bifidobacteria [57,58,59]. The gene *pkb2* encoding the Pkb2 protein kinase exhibited the smallest homology between different bifidobacterial species (43–77%, BLASTP) and the highest homology among strains of the same species (98–100%). Thus, Pkb2 qualified as a species-specific STPK. The genes in the neighborhood of pkb2 were also found to be highly conserved and included a species-specific cluster of linked genes that were unique to bifidobacteria [54]. The PFNA cluster was detected in 71 out of 86 species of bifidobacteria, which typically inhabit various ecological niches including the GIT of mammals, birds and insects. The genes of the PFNA cluster, which vary between five and eight depending on the species are transcribed in one direction (Figure 1). Since the PFNA cluster was experimentally shown to function as an operon only in the genus *B. longum *subsp.* longum* GT15, we continue to refer to it as a cluster until it is proven to function as an operon in other species.

**Figure 2:**
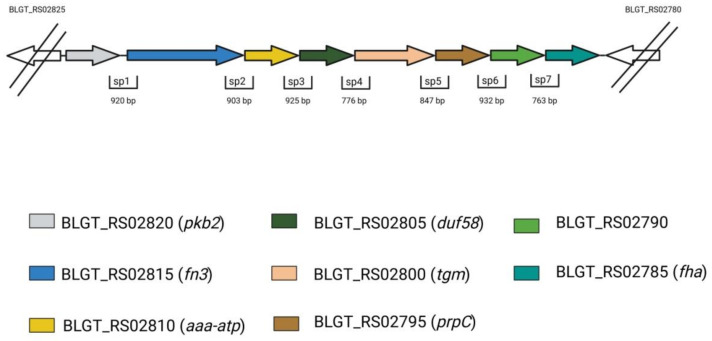
Transcriptional organization of the PFNA operon in *B. longum *subsp.* longum* GT15 [61]. Adapted from [60].

The genes in the PFNA cluster are always positioned in the same order, with the first five being common to all bifidobacterial species. These are *pkb2, fn3, aaa-atp, duf58* and *tgm*, and they encode the proteins Pkb2, a protein containing a fibronectin type III domain (FN3), AAA-ATPase (*aaa-atp*), a hypothetical protein with a DUF58 domain and a TGM, respectively. As for the remaining three genes, the sixth (protein phosphatase, *prpC*), the seventh (hypothetical protein, BLGT_RS02790) and the eighth (protein containing the FHA domain, *fha*), they were only found in certain species (Figure 1). Clusters containing all eight genes were only found in the species *B. longum*, *B. bifidum*, *B. saguini*, and *B. aesculapii*. The median sequence identity for these proteins was very low between species, which indicates that all these proteins including Pkb2 are highly divergent [54,57]. This protein sequence divergence was shown to be driven by positive selection, which was likely exerted by the host’s immune system [23]. The structural conformity of the cluster across various bifidobacterial species, which extends to the gene products, suggested that the cluster could function as an operon. This hypothesis was proven right when all eight genes of the cluster were shown to be co-transcribed in *B. longum *subsp.* longum GT15* (Figure 2). The transcription start site for this polycistronic mRNA was located 40–41 bp. upstream of the start codon *pkb2* (Figure 2). An additional transcription start site was found in the putative operon within 58–61 bp. upstream of the start codon *prpC*, which possibly implies a complex regulation of gene transcription. The putative transcription terminator was predicted using in silico methods within 65 bp. downstream of the fha reading frame stop codon (Figure 2). Taken together, these data confirm that the genes making up the PFNA cluster are organized in an operon [54]. It is also important to point out that the genomic localization of the PFNA cluster differs between phylogenetic groups of bifidobacteria.

The function of the operon was inferred from the known functions of its genes. As noted earlier, the *pkb2* gene encodes a eukaryotic type STPK that was predicted to contain a catalytic domain and a transmembrane alpha-helix. STPKs normally comprise one-component sensory signaling systems. The function of STPK involves binding ligands and triggering intracellular signaling through reversible phosphorylation of substrates. The C-terminal region of the STPK Pkb2, known for its role in processing external stimuli, is highly variable among species. The structural differences of this region, even in closely related species belonging to the same phylogroup, may indicate that different bifidobacterial species can bind different ligands [23]. However, more conclusive evidence is needed to ascertain whether components of the host immune system can be ligands for Pkb2. STPKs directly modulate transcription in many bacteria by responding to specific external signals. Previously, 12 molecules were identified as phosphorylation substrates for Pkb2 [54]. Eleven substrates that were phosphorylated in the presence of Pkb2 were functionally classified as transcription and translation proteins, proteins belonging to the F1 region of the F0F1-ATPase, ABC transporters, a molecular chaperone and glutamine synthase (GlnA). All these proteins are considered moonlighting proteins and ostensibly can enable bifidobacteria to adhere to the intestinal epithelium of the host organism and to interact with the immune system and other cells of the host organism [61,62]. The phosphorylation substrate Pkb2 is the MoxR chaperone belonging to the the AAA+ATPase family, which is encoded by the *aaa-atp* gene of the PFNA operon. Pkb2 is capable of phosphorylating the serine, threonine, and tyrosine residues of proteins. The *aaa-atp* gene encodes an AAA+ATPase that belongs to the MoxR family. AAA+ATPases contain a number of conserved sequence motifs involved in ATP binding and hydrolysis [63]. These proteins are usually oligomers and often form hexameric rings. Although they are not usually found in eukaryotes, proteins of the MoxR family of AAA+ATPases are widespread across bacteria and archaea. ATPases of the MoxR family are important chaperons that regulate assembly, maturation and activation of specific multimeric protein complexes involved in vital processes such as metabolism, cell growth and development, tolerance to various types of stress and pathogenesis [64,65]. More specifically, the studied ATPase belongs to the MoxR Proper (MRP) subfamily. AAA+ATPase is a phosphorylation substrate of the protein kinase Pkb2.

The *fn3* gene encodes a large secreted glycoprotein that contains motifs of cytokine receptors within FN3 domains. The functions of FN3 are related to adhesion and interaction with the host’s immune system achieved by binding cytokines [22].

**Figure 3:**
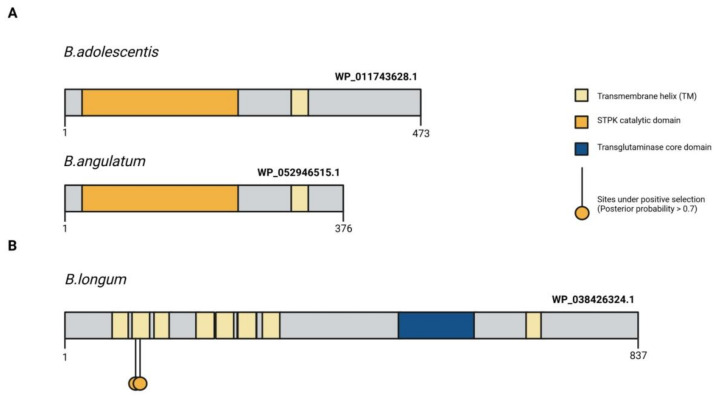
Protein structure of the signal-transduction domains of the PFNA operon potentially involved in interaction with the host organism. (**A**) Comparison of the primary structure of STPK Pkb2 between *B. angulatum* and *B. adolescentis* species both belonging to the *B. adolescentis* phylogroup. Pkb2 contains a catalytic kinase domain, a transmembrane domain (TM), and a C-terminal signal region [23]. (**B**) Localization of amino acid sites under pervasive positive selection in all branches of the bifidobacterium phylogenetic tree in the primary structure of the WP_038426324.1 protein encoded by the *tgm* gene in *B. longum *subsp.* longum GT15* genome. The figure shows the domain organization of the protein, as well as the localization of candidate sites with a PP value > 0.7 [23]. Adapted from [23].

The function of the DUF58 domain, which is part of a protein encoded by the *duf58* gene, is unknown. The *tgm* gene encodes a transglutaminase-like enzyme, presumably a cysteine protease, which appears to be involved in signal transduction. Episodic positive selection might have led the proteins encoded by the *tgm* and *pkb2* genes to become species specific signaling molecules [22]. Figure 3 illustrates the structure of the TGM protein containing exposed transmembrane domains that potentially interact with host ligands. The *prpC* gene encodes a serine-threonine protein phosphatase. This enzyme performs reversible dephosphorylation of protein substrates and works in conjunction with a serine-threonine protein kinase. The function of the seventh gene and its product is unknown. The last gene of the cluster is *fha* and it encodes a protein containing the FHA domain. FHA domains are found in eukaryotic and prokaryotic proteins alike and can bind to phosphotreonine, phosphoserine, and sometimes phosphotyrosine. They are involved in the development and maintenance of cell cycle checkpoints, DNA repair and transcriptional regulation [66]. It was shown that episodic positive selection amino acid sequence evolution of the proteins making up the species-specific PFNA operon. Since the *fn3* gene encodes a protein containing two type III fibronectin domains, which include cytokine receptor motifs, it is likely that the function of the PFNA cluster is associated with species-specific communication between most bifidobacterial species and the host immune system [22].

## 4. FN3: Species Specificity, Divergence, Structure, Function

The domains of FN3 are widespread among animal and human proteins. The domains of FN3 have been found in many protein families: extracellular matrix molecules, cell surface receptors, enzymes, and muscle proteins. Apart from being an evolutionarily conserved domain, it participates in a panoply of cellular functions: cell adhesion, migration, growth, and differentiation [67]. The domains of FN3 are found in both extracellular and intracellular proteins and it contains a conserved beta sandwich fold with one beta sheet containing four strands and another sheet containing three strands. Despite a lack of an evolutionarily relation between the two, the fold is topologically very similar to the fold of Ig-like domains, [68]. The 3D structures of human FN3 domains have been well studied [68] and the effect of extracellular phosphorylation on its function and interaction with other proteins has been shown [69].

The domains of FN3 are important extracellular components of human type I cytokine receptors that play an important role in binding cytokines [70,71]. One of the distinctive features of type I cytokine receptors is the type III fibronectin domain containing a WS-WS consensus motif (WS-motif, cytokine receptor motif, any amino acid in the middle of the motif) [72]. This motif plays a role in receptor folding, cytokine binding and signal transduction [73]. The results of computer modeling suggest that the region of interaction between interleukins and type I cytokine receptors is adjacent to the WS-WS motif [74]. Mutations in this motif alter the binding affinity between the receptor and the cytokine [72].

Over recent years, the 10th FN3 domain of the human fibronectin has been actively employed for the development of alternative scaffold proteins (ACPs) capable of high-affinity binding of antigens [75]. These proteins are often used for engineering synthetic binding proteins due to their high stability, low molecular weight and are easy to produce using bacterial expression systems. Some view this approach as a replacement for antibody therapy [76,77]. For example, ACPs have been developed based on the 10th FN3 domain of human fibronectin for binding the cancer biomarker mesothelin [78]. ACPs are also being actively developed for binding one of the main mediators of inflammation and innate immunity, TNF-α [79].

The domains of FN3 are also found in yeasts, plants, and bacterial proteins, especially in those derived from gut-dwelling bacteria such as Lactobacillus, Clostridium, Bacteroides, Enterococcus, *Escherichia coli*, including the human pathogens *Helicobacter pylori*, *Mycobacterium tuberculosis*, *Klebsiella pneumonia*, *Pseudomonas aeruginosa*. Bacterial FN3 domains are found in functionally eclectic proteins, namely cellulases, hydrolases and glucosidases, where they fulfill a structural and enzyme stabilizing function [79]. There are accounts of total inactivation of enzymes resulting from deletion of the FN3 domain [80].

**Figure 4:**
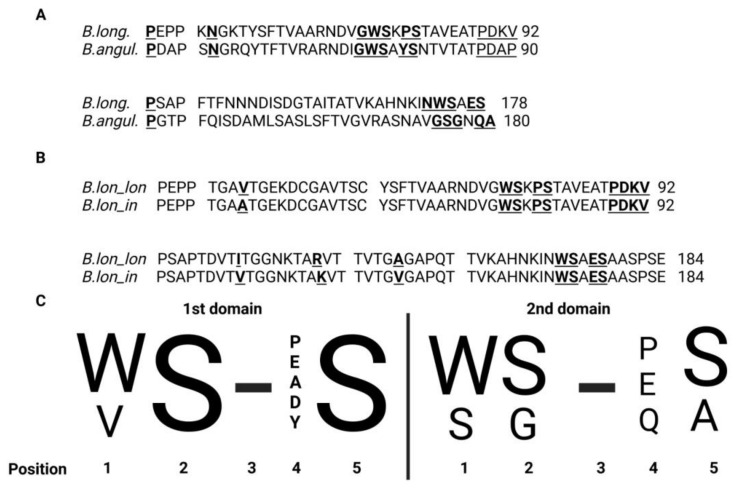
(**A**) Alignment of sequences of protein fragments containing two FN3 domains derived from *B. longum GT15* and *B. angulatum* GT102 (identity 40.1%; similarity 68.7%). Cytokine motifs in these species of bifidobacteria exhibited the starkest differences. The amino acids interjected between the domains are underscored. Amino acids in the “Cytokine receptor motif” site are highlighted by a double under-score. Amino acids in the “Interdomain contacts” site are highlighted in bold and underlined. (**B**) Alignment of sequences of protein fragments containing two FN3 domains from *B. longum *subsp.* longum* and *B. longum *subsp.* infantis* (identity 97.8%; similarity 100%). Differences in amino acid sequences in the first domain of FN3: 43 V → A; in the second domain FN3: 101 I → V, 109 R → K and 132 A → V. The amino acids interjected between the domains are underscored. Amino acids in the “Cytokine receptor motif” site are highlighted by a double under-score. Amino acids in the “Interdomain contacts” site are highlighted in bold and underlined. (**C**) This logo diagram resulted from aligning the corresponding aminoacid sequences of the gut-dwelling species *B. longum*, *B. adolescentis*, *B. bifidum*, *B. breve*, *B. catenulatum*, *B. pseudocatenulatum*, *B. dentium*, *B. angulatum*, *B. kashiwanohense* and *B. gallicum*. The motif in the second domain marked by an arrow is unique to *B. angulatum*. Any amino acid can be in position 3. Adapted from [60].

Recently, a unique FN3 protein encoded by one of the genes of the PFNA operon, the *fn3* gene, was discovered in bifidobacteria. This protein was found to be unique to bifidobacteria and contained two functional domains. FN3 is a secreted protein that contains a transmembrane domain [23,62,81]. FN3 presumably plays a role in the adhesion of bifidobacteria to the intestinal epithelium of humans. However, the cytokine receptor motifs in two of the protein domains suggest that FN3 may act as a receptor for binding host-derived cytokines. Sequence alignment of the motifs revealed significant differences in two species that stood out from others (Figure 4A). The first species *B. longum* (WS-PS and WS-ES) typically lives in the human intestinal microbiota, whereas the second species *B. angulatum* is rarely isolated from humans (WS-YS and SG-QA) (Figure 4C). This may be explained by interactions with various cytokines (Figure 3) and by variation in adaptability to the human body [81].

Table 1 illustrates the motifs of cytokine receptors found in the fibronectin domains of FN3 proteins belonging to distinct phylogenetic groups of bifidobacteria [8]. Except for *B. cuniculi* and *B. gallicum* containing the motifs DS-WS and VS-PS, respectively, the sequence motifs of cytokine receptors of the first FN3 domain WS-PS, WS-ES, WS-DS, WS-AS, WS-YS, and WS-SS were highly conserved among bifidoabcterial species. The classification featured in Table 1 is based on the aligned sequences of the motifs of cytokine receptors from the second FN3 domain. Class I is formed by conserved cytokine receptor motifs identical to motifs of the first domain. Classes II–VI contain sequences of cytokine receptor motifs that are distinct from the motifs of the first and second domains of the first class. Species missing the second FN3 domain altogether fall into classes VII and VIII.

Phylogenetic groups of bifidobacteria often correlate with specific ecological niches and different animals [8]. For instance, members of the *B. tissieri* group are commonly found in monkeys [82], members of the *B. pullorum* group in chickens [8], members of the *B. bombi* inhabit the intestines of bumblebees [83], members of the *B. psychraerophilum* group are often isolated from dairy products [84,85] and members of the *B. boum* are typical residents of livestock [8]. A correlation was established between these groups of closely related species of bifidobacteria, the ecological niches they inhabit, and the type of cytokine receptor motifs harbored by them. More specifically, members of the *B. tissieri* group typically contain the motifs WS-PS and DG-EG/EA, members of the *B. bombi* group are often marked by the motifs WS-PS and DG-VS, and members of the *B. boum* group feature the motifs WS-ES and WS-PS. Moreover, members of the groups *B. pullorum* and *B. psychraerophilum* completely lack the PFNA cluster and the *fn3* gene encoding the FN3 protein. Larger groups, which comprise cosmopolitan members capable of inhabiting various species of animals and humans, are described by greater variety of sequence motifs of cytokine receptors and conserved sequence motifs are less common. For example, in members of the groups *B. asteroides*, *B. bifidum* and *B. pseudolongum,* only the motifs of the first fibronectin domain are conserved. The motifs of the second fibronectin domain are highly diverse and sometimes the domain is absent altogether as in the case of members of the *B. pseudolongum* group. In members of the *B. adolescentis* group, only the second FN3 domain is conserved. The most diverse sequence motifs of cytokine receptors of both FN3 domains are exhibited by members of the *B. longum* group, which are found in various animals and humans. Interestingly, human-derived bifidobacterial species, which belong to different phylogenetic groups of bifidobacteria, display strong differences in sequence motifs. Hence the hypothesis that the noted differences in sequence motifs between species mirror the differences in the host immune status and are the result of competition that unraveled in the intestines of host organisms. There is great interest in studying how variations in sequence motifs of cytokine receptors and fibronectin domains (Figure 4A) affect interaction with the host immune system. Certain SNPs in fibronectin domains might account for the intra-strain differences within each species and the adaptive advantage conferred by them. For example, the subspecies *B. longum *subsp.* longum* and *B. longum *subsp.* infantis* differ from one another only by four amino acid substitutions (Figure 4B).

## 5. Binding of the FN3 Protein Fragment to Cytokines (TNF-α)

Cytokines play a major role in the immunopathology of viral infections. A rapidly developing and well-coordinated innate immune response is the first line of defense against viral infections. However, an out-of-control and excessive immune response can have opposite effects on health [86]. Sometimes a disproportionate number of cytokines is produced spilling over into a so-called cytokine storm [87]. Such an immune response is dangerous and can be fatal for the body. Cytokine storms are one of the possible outcomes of COVID-19 [88]. At the same time, pathogenic bacteria can bypass the body’s defense systems using special proteins that serve as cytokine traps for tuning down inflammation. Scientists have been wondering whether bifidobacteria, as intestinal symbionts, can impact inflammation in the same way. It turned out that bifidobacteria have the potential to alleviate “cytokine storms” due to a heterogeneous effect on pro-inflammatory and anti-inflammatory cytokines [88]. However, the mechanism underlying these effects has not been studied.

In bifidobacteria, the FN3 surface protein has two fibronectin domains that form an Ig-like fold similar to those found in human cytokine receptors [67]. A recent study set out to find out whether this protein is capable of blocking or binding cytokines. The authors used a fragment of the FN3 protein containing two fibronectin domains that isolated from *Bifidobacterium longum *subsp.* longum GT15*. To detect specific interaction between the FN3 protein and cytokines, a sandwich ELISA was devised [22]. FN3-specific antibodies were first attached onto the surface of the wells of a polysterol plate. Then, a fragment of the FN3 protein was added and followed by cytokines and specific fluorescent labeled antibodies. As a result, the ELISA sandwich had the following structure: antibody–FN3–cytokine–antibody. Of the four tested cytokines TNF-α, IL-6, IL-1β and IL-10, effective binding was observed only for TNF-α, which is one of the main triggering factors of cytokine storms. The fact that surface proteins of bifidobacteria can recognize and bind certain classes of cytokines provides sufficient evidence for that mechanism being one of bifidobacteria’s pathways of immunoregulation. It has been presumed that other species of bifidobacteria sustain cytokine homeostasis in the enteric immune system by binding other cytokines such as IL-6 and IL-beta. A recent study demonstrated how increasing concentration of TNF-α in the growth medium affects gene expression in bifidobacteria [89].

**Figure 5:**
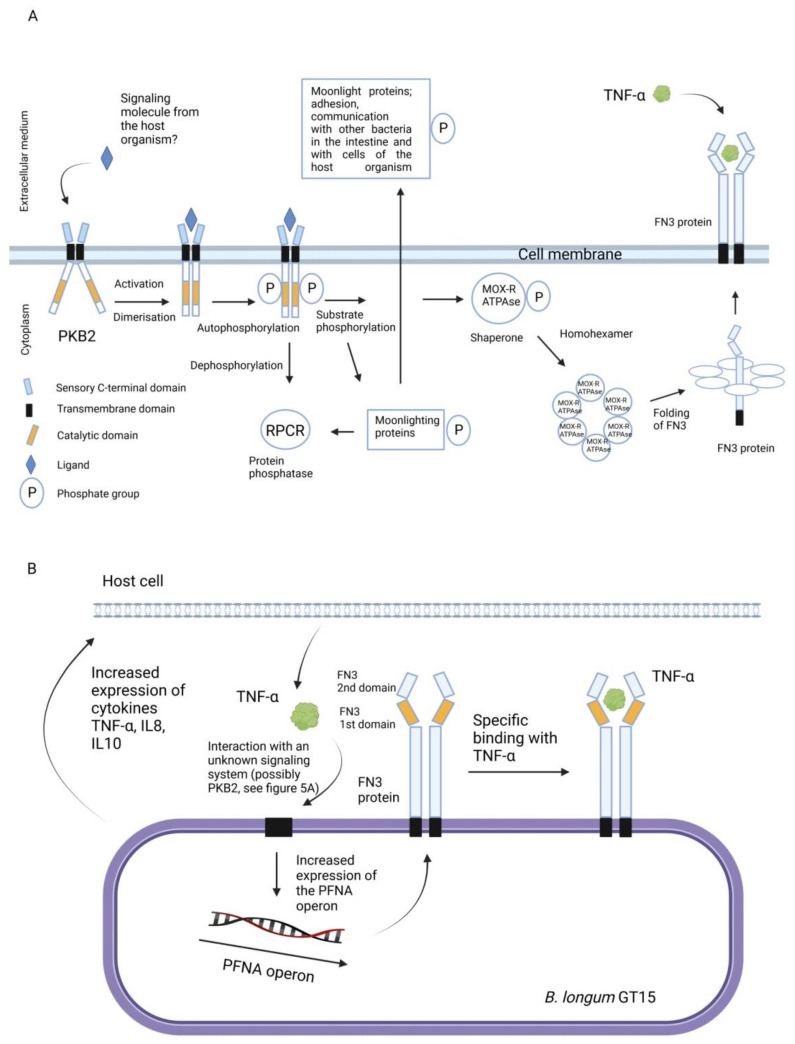
Hypothetical schematic representation of the role of the PFNA operon in the interaction of bifidobacteria with the host immune system. (**A**) Activation of the PKB2 signaling system. The serine-threonine protein kinase (PKB2), following its activation by an unknown ligand, autophosphorylates, [57] and phosphorylates other substrates. The phosphorylated substrates are moonlighting proteins [54], which participate in the adhesion and interaction of bifidobacteria with cells of the host organism. PKB2 also phosphorylates a protein of the PFNA operon, the MoxR-ATPase protein encoded by the *aaa-atp* gene [54]. MoxR-ATPase is a chaperone and thus is possibly involved in folding of proteins, including, FN3. The FN3 protein is capable of specifically binding to TNF-α [22]. (**B**) The strain *B.longum* GT15 increases the expression of cytokines TNF-α, IL8 and IL10 in human cells [89]. Apparently, TNF-α interacts with an unknown signaling system of *B. longum* GT15 (possibly PKB2), triggering an increase in the expression of genes of the PFNA operon, including the *fn3* gene [89]. Adapted from [60].

Using whole transcriptome analysis, the authors demonstrated that supplementation of *B. longum GT15* culture medium with high concentration of the pro-inflammatory cytokine TNF-α (10 ng/mL) altered the expression of 1000 genes and 176 operons [89]. Many of the genes whose expression was significantly increased, among which was *fn3*, encoded proteins with antioxidant activity such as heat shock protein (hsp20) [90], glutathione [91], ABC transporters [92], enzymes involved in the metabolism of amino acids such as leucine, arginine [87], and short-chain fatty acids including propionate [93]. The role of the PFNA operon and the proteins encoded by it in the interaction of bifidobacteria with the host immune system have not been studied in detail. At this stage, we experimentally identified some of the elements involved in this interaction. Figure 5A,B illustrates two hypothetical diagrams describing these mechanisms that are partially based on experimental data. Further research and preclinical studies are needed to fully understand these mechanisms.

If we assume that TNF-α, which specifically binds to FN3, may be an unknown ligand interacting with PKB2, then the two presented diagrams can be combined into one reflecting the bi-directional interaction between bifidobacteria and the host, involving the PFNA operon.

It is especially important to understand how bifidobacteria, the prevalent genus in newborns, shape the latter’s immune system. The authors also conjectured that a fragment of the FN3 protein that binds to TNF-α will contribute to reducing the effects of cytokine storms in severe COVID-19 patients. To say the least, the fibronectin type III domain and certain bifidobacterial strains could bolster the existing approaches of immunotherapy of inflammatory diseases including COVID-19.

## 6. TNF-α, IL-6 and Regulation of Cytokine Storms

Stimulation of the immune system excessive in intensity and duration results in an increase in the production of cytokines at the site of inflammation. Disproportionate responses such as “cytokine storms” entail the following outcomes: (1) mobilization of cell-mediated and humoral immunity at the site of inflammation, which is necessary to fight infection, (2) damage to surrounding tissues, (3) systemic inflammation and sepsis in the most severe cases. In the event of a viral infection, for instance in viral pneumonia, the main mediators of cytokine storms are IL-6, TNFα, IL-8, IFNg, chemokines secreted by T-lymphocytes, monocytes, tissue-resident macrophages, neutrophils, fibroblasts, endothelial cells and tissue-forming cells such as alveolocytes [94,95].

IL-6 is one of the key contributors to virus-induced cytokine storms. IL-6 acts by binding to the membrane-bound IL-6 receptor (IL-6R) and forming a complex with the membrane glycoprotein gp130. The latter step triggers a Jak/STAT and NFkB-mediated pro-inflammatory response in target cells: macrophages, neutrophils, T- and B-lymphocytes [96]. TNF-α activates immune cells through two types of receptors, TNFR1 and TNFR2. TNFR1 induces mainly a pro-inflammatory profile in cells and enhances transendothelial migration of lymphocytes. The mechanism of action of TNFR2 is related to regulation of anti-inflammatory cytokines [97]. In SARS-CoV2-associated disorders, TNF-α interacts with interferon gamma activating the transcriptional activity of IRF1 and STAT1 and causing extensive cell death and substantial tissue damage [95].

The undirected effects of IL-6 and TNF-α on non-immune cells are typically observed in cytokine storms. As IL-6 levels soar, they begin forming soluble complexes with IL-6R, which in turn bind to gp130 on the surface of cardiomyocytes [98], endothelial cells (where typical pro-inflammatory activation occurs) [99], neurons [100,101] and, possibly, other cell populations. These interactions set off Jak/STAT, MAPK, PI3K, and Notch signaling cascades [96]. As for the TNF-α receptors TNFR1 and TNFR2, their expression is not restricted to immune cells extending to neurons, cardiomyocytes and endothelial cells [102]. TNFR1—belonging to a family of death receptors—once activated, can initiate apoptotic death of target cells via activation of the Fadd and caspase 8 proteins. Contrastingly, TNFR2 promotes cell survival.

The detrimental effects of IL-6 and TNF-α on neurons could be summed up as an increase in the activity of Na and Ca channels, excessive excitation and neurotoxicity [101,103]. All the while, TNFR2 signaling was shown to protect neurons from oxidative stress [104]. It is also noteworthy that the pro-inflammatory microenvironment activates TNFR2 expression and induces an anti-inflammatory profile in microglia [105]. In cardiomyocytes, IL-6-mediated signaling can become a major cause of cell hypertrophy as a consequence of excessive activation of ion channels. This condition is further reinforced by fibroblasts, which ultimately leads to ventricular or atrial fibrillation [106,107]. Low levels of TNF-α act beneficially on cardiommyocytes and yield protective effects via Nrf2 activation [108]. However, an increase in TNF-α levels activates the transcription factor NFkB stimulating inflammasome formation and cell death, another cause of arrhythmia [109]. This contrast can probably be explained by the alternating selective protective properties of TNFR2 and the proapoptotic effects of TNFR1. Overall, both IL-6 and TNF-α are major contributing factors to the development of cytokine storms as well as neurological and cardiovascular pathologies characteristic of viral infections such as COVID-related medical conditions, and therefore they are important targets for immunomodulatory therapy.

Today, the main therapeutic targets of anti-IL-6 and anti-TNFα therapy are (1) IL-6 and its receptors—membrane-bound and soluble form—as well as the membrane glycoprotein gp130; (2) TNF-α and its receptor TNFR1, which exhibits a pro-inflammatory and cytotoxic activity. Currently, monoclonal antibodies against IL-6, IL-6R and TNF-α have been approved for use. Monoclonal antibodies targeting TNFR1 are undergoing clinical trials (Table 2). Gp130 is unlikely to become a target since it participates in relaying signals from other molecules such as the anti-inflammatory cytokine IL-11, the neurotrophic factor CNTF, and the antitumor agent LIF [110].

Whether the FN3 type-III domains of bifidobacteria will be adopted in the immunotherapy of inflammatory diseases and the regulation of the dysbalanced immune system characteristic of COVID-19 [122] will be shown by future studies. One thing is certain, FN3 fibronectin domains containing cytokine motifs represent a large natural arsenal of molecules that evolutionarily adapted to the host organism. This is their main advantage over libraries of artificially engineered combinations based on the 10th FN3 human fibronectin domain. Monoclonal antibodies-based treatments against pro-inflammatory cytokines are known for their infamous side effects [123]. Bifidobacteria, which are known for their anti-inflammatory properties, might be a better substitute [36,37,38]. Pharmacological components of bifidobacteria named postbiotics, which the FN3 protein, are another exciting area of research fueling antiviral drug development. In light of these findings, studying the immunomodulatory and antioxidant properties of bifidobacteria isolated from bats and other possible sources of coronaviruses, primarily SARS-CoV2, might increase our chances of discovering bifidobacterial strains with unique properties.

## 7. Challenges and Limitations

The potential of FN3 lies in the treatment of immune-related complications observed in diseases of various etiology. The selective binding, which occurs between the *B. longum GT15*-derived FN3 protein and TNF-α, indicate that this protein is a promising candidate for the regulation of TNF-α levels in various organs and tissues. Similar drugs are being used today in the immunotherapy of autoimmune, oncological and viral diseases. Drawing on the high level of expression in *E. coli* and the possibility of generating sufficient amounts of the protein in the native state, one advantage to developing drugs based on the FN3 protein will be a relatively low cost compared to monoclonal antibodies. The second putative advantage is most likely the absence of addiction to the drug or any serious side effects due to the familiarity of the immune system with the protein. The most important questions related to the proteins’ immunogenicity, stability, efficacy, dosage and mode of delivery will have to be addressed in preclinical and clinical studies. At this stage, it is likely that nanocapsules as a method of delivery will be a viable solution for the FN3 protein fragment.

## 8. Perspectives

The PFNA operon of bifidobacteria is a unique gene cluster that is most likely the product of selective evolutionary pressure. This is evidenced by the high adaptive genetic divergence in various species of bifidobacteria and the conservatism of gene sequences within species. Moreover, the PFNA operon contains from five to eight genes depending on the species, which could imply the operon’s involvement in the process of bacterial speciation. Replacement of some genes of the PFNA cluster of one species with those of another may be of great interest for studying experimental evolution using bifidobacteria as a model.

It is a fundamental task in evolutionary studies of bacteria to identify similar operons involved in speciation in other groups of bacteria of the human microbiome. Three genes of the PFNA operon, to say the least, *pkb2*, *tgm* and *fn3*, are apparently capable of interacting with ligands of the host and perhaps other bacteria. The ligands in question are yet to be determined.

Since the FN3 domain of *B. longum* GT-15 selectively binds to TNF-α, it is one of the key genes of the PFNA operon. Significant differences in the amino acid composition of the FN3 domain among different species of bifidobacteria suggests a possible interaction with pro-inflammatory and anti-inflammatory cytokines in other species. Comparison of the FN3 domain 3D structures of different species should be conducted at this stage. Site-directed mutagenesis of critical domains might be employed to generate new structures with new functions.

The FN3 domains of bifidobacteria are an exciting field of research for the treatment and prevention of immune disorders, not only in the context of virus-induced cytokine storms, but to be used along with monoclonal antibodies to bolster the therapy of chronic diseases accompanied by inflammation such as cancer. Bifidobacteria and postbiotics based on them have already proven effective in these areas.

## 9. Conclusions

Bifidobacteria are obligate anaerobic bacteria that inhabited Earth long before the atmosphere’s oxygenation. As the percentage of oxygen in the atmosphere surged, bifidobacteria’s potential habitats became limited to anoxic niches such as the intestines of animals and others. Bifidobacteria are credited with shaping the immune system of animals ranging from insects to humans. The genus *Bifidobacterium* encompasses 86 species and 13 subspecies that are found in the intestines of many members of the animal kingdom. One of the species considered human in origin, *B. longum *subsp.* infantis*, is the first to colonize the intestines of newborns actively building up their immunity. Bifidobacteria induce the expression of cytokines via specialized toll-like receptors as shown in rodent and human cell lines. Yet, it remains largely unknown how symbiotic bacteria such as bifidobacteria communicate with the host’s immune system. Grounded in the assumption that bifidobacteria must have inevitably developed species-specific genes enabling them to communicate with their hosts, recent studies set out to identify genes or groups of genes responsible for specific interactions with components of the host’s immune system. It was presumed that such an interaction had to be species-specific and the responsible proteins must contain signal-transducing elements. A species-specific gene encoding the signal-transducing element, serine-threonine protein kinase Pkb2, was found in most species of the genus Bifidobacterium. The Pkb2 gene is part of a gene cluster designated PFNA. The PNFA cluster is present in most bifidobacterial species containing five to eight genes, and all of them show high divergence between species, which is the result of positive selection. One of the key genes of the cluster is the *fn3* gene, which encodes a protein containing two type III fibronectin domains that include cytokine receptor motifs. After careful examination of the ability of the FN3 protein fragment from *B. longum *subsp.* longum* GT15 to bind human cytokines (TNF-α, IL-6, IL-1β, IL-10) it has been established that FN3 specifically binds to TNF-α. It was found that when growing *B. longum *subsp.* longum* GT15 in a medium containing TNF-α, the transcription level of 176 operons, including those responsible for the antioxidative properties of the strain, changes, and the expression of genes of the PNFA operon, including the *fn3* gene, is enhanced. Studies are being conducted to better understand the molecular mechanisms underlying the binding of the FN3 protein to the interleukin TNF-α.

The data gathered in this study support the hypothesis that bifidobacteria employ the PFNA cluster to communicate with components of the human immune system. At the very least, two genes of the PFNA operon, *pkb2* and *tgm*, encode proteins that participate in signaling by binding to their corresponding ligands. These genes encode a sensory and variable transmembrane region of the serine-threonine protein kinase PKB2 and the transmembrane region of a TGM, respectively [23,24]. It is also possible that the PFNA cluster is used by bifidobacteria to communicate with each other. Further research into the PFNA cluster could improve our understanding of speciation in bacteria driven by adaptation to the host organism. Similarly, the study of molecular tools of communication evolved by other bacterial genera and families, warrants scientific attention. The PFNA cluster in the subspecies *B. longum subsq longum* and *B. longum subsq infantis* could be used as a starting point for studying these processes [11,51]. Another way to approach the question of how mutations in the PFNA operon affected the adaptive potential of bifidobacteria is to compare this operon in bifidobacteria isolated from different domesticated animals [107].

It is highly important to understand how exactly TNF-α interacts with the FN3 protein and what is the role of cytokine motifs and the putative beta-sandwich structure. It is likely that FN3 proteins from other bifidobacterial species can selectively interact with other pro-inflammatory (IL-6, IL-1β) and anti-inflammatory cytokines (IL-10). In recent years, research was invested in the 10th human fibronectin type III domain as a prospective anti-inflammatory drug. FN3 bifidobacteria-derived proteins can contribute significantly to the expansion of this line of research. After all, FN3 proteins are synthesized by probiotic bacteria, they adapted during evolution to the human immune system, and they are involved in maintaining homeostasis.

Furthermore, the study of the cytokine-binding properties of proteins of microbial origin is a pressing issue given the current epidemiological situation. One of the most severe outcomes of COVID-19 is uncontrolled inflammation, otherwise known as cytokine storm. Selective binding of TNF-α, one of the key factors of inflammation, by the FN3 protein fragment of *Bifidobacterium longum* opens up prospects for the development of new drugs aimed at keeping this cytokine in check in target diseases.

The year 2021 marks the 150th anniversary of Charles Darwin’s publication of theories of human evolution [124], a turning point in history that laid the foundation for countless scientific discoveries including bifidobacteria and their role of evolution of humans and animals.

## Figures and Tables

**Table 1:** Motifs of cytokine receptors found in the fibronectin domains of bifidobacteria belonging to different species and phylogenetic groups.

Species	Source of Isolation	Motifs of Cytokine Receptors		Class	Phylogenetic Group According to [8]
		1st FN3 Domain	2nd FN3 Domain		
***B. adolescentis***	Human	WS-DS	WS-PS	I	***B. adolescentis***
***B. catenulatum***_***catenulatum***	Human	WS-ES	WS-PS	I	
***B. catenulatum***_***kashiwanohense***	Human	WS-ES	WS-PS	I	
***B. dentium***	Human	WS-PS	WS-PS	I	
***B. moukalabense***	Monkey	WS-PS	WS-PS	I	
***B. pseudocatenulatum***	Human	WS-ES	WS-PS	I	
***B. ruminantium***	Cow	WS-ES	WS-PS	I	
***B. boum***	Cow	WS-ES	WS-PS	I	***B. boum***
***B.*** *** porcinum***	Pig	WS-ES	WS-PS	I	
***B. thermacidophilum***	Soy whey	WS-ES	WS-PS	I	
***B. thermophilum***	Pig	WS-ES	WS-PS	I	
***B. pullorum_gallinarum***	Chicken	Both the PFNA cluster and the gene encoding FN3 are absent			***B. pullorum***
***B. pullorum_ saeculare***	Rabbit				
***B. pullorum_ pullorum***	Chicken				
***B. asteroides***	Bee	WS-AS	SG-VA	II	***B. asteroides***
***B. actinocoloniiforme***	Bumblebee	WS-PS	SG-AA	II	
***B. coryneforme***	Bee	WS-AS	AG-AR	III	
***B. indicum***	Insect	WS-AS	AG-AR	III	
***B. longum***_***infantis***	Human	WS-PS	WS-ES	I	***B. longum***
***B. longum***_***longum***	Human	WS-PS	WS-ES	I	
***B. longum***_***suis***	Pig	WS-PS	WS-ES	I	
***B. breve***	Human	WS-AS	WS-ES	I	
***B. aesculapii***	Monkey	WS-ES	DG-SA	IV	
***B. stellenboschense***	Monkey	WS-DS	DG-GA	IV	
***B. angulatum***	Human	WS-YS	SG-QA	II	
***B. merycicum***	Cow	WS-YS	SG-QA	II	
***B. psychraerophilum***	Pig	The gene encoding FN3 is absent			***B. psychraerophilum***
***B. aquikefiri***	Kefir				
***B. crudilactis***	Milk				
***B. bifidum***	Human	WS-PS	EG-PS	I	***B. bifidum***
***B. biavatii***	Monkey	WS-PS	VG-HG	V	
***B. scardovii***	Blood	WS-PS	DG-PG	IV	
***B. animalis***_***animalis***	Rat	WS-DS	AS-PS	III	***B. pseudolongum***
***B. animalis***_***lactis***	Milk	WS-DS	AS-PS	III	
***B. pseudolongum***_***pseudolongum***	Pig	WS-SS	TG-PS	VI	
***B. pseudolongum***_***globosum***	Cow	WS-SS	TG-PS	VI	
***B. choerinum***	Pig	WS-SS	Domain absent	VII	
***B. cuniculi***	Rabbit	DS-WS	Domain absent	VII	
***B. gallicum***	Human	VS-PS	Domain absent	VIII	
***B. bombi***	Bumblebee	WS-PS	DG-VS	IV	***B. bombi***
***B. commune***	Bumblebee	WS-PS	DG-VS	IV	
***B. tissieri***	Marmoset monkey	WS-PS	DG-EA	IV	***B. tissieri***
***B. vansinderenii***	Bare-faced marmoset	WS-PS	DG-EA	IV	
***B. catulorum***	Marmoset monkey	WS-PS	DG-EG	IV	
***B. primatium***	Bare-faced marmoset	WS-PS	DG-EG	IV	

**Table 2:** Monoclonal antibodies approved for anti-IL6 and anti-TNFα therapy.

Name of the Drug	Target	Origin	Target Disease	Other Trade Names	Results of Anti-COVID-19 Trials	References
Tocilizumab	IL6R	Recombinant humanized monoclonal antibody	CRS Rheumatoid arthritis Giant cell arteritis Juvenile idiopathic arthritis CAR T cell induced cytokine storm	Actemra RoActemra	Insufficient evidence of efficacy	[111]
Sarilumab	IL6R	Human IgG1 monoclonal antibody	Rheumatoid arthritis	Kevzara	Unknown, preliminary results are positive	[112]
Левилимаб/Levilimab	IL6R	Human monoclonal antibody	Rheumatoid arthritis	Ilsira (Biocad)	Unknown, approved for clinical trials	[113]
Siltuximab	IL6	Human–murine chimeric monoclonal antibody	Multicentric Castleman disease; CAR T cell induced cytokine storm	Sylvant	Unknown, a candidate	[114,115]
Infliximab	TNFα	Chimeric murine/human IgG1	Rheumatoid arthritis, ankylosing spondylitis, Crohn’s disease and ulcerative colitis, psoriasis and psoriatic arthritis	Remicade	Unknown, undergoing clinical trials (1) a positive effect; (2) mild formation of antibodies against the virus is observed; inflammation and multisystem dysfunction	[116,117,118,119]
Adalimumab	TNFα	Fully Human IgG1	Autoimmune inflammatory diseases, including rheumatoid arthritis, Crohn’s disease, and psoriatic arthritis	Humira	Clinical trials are scheduled	[119]
Certolizumab-pegol	TNFα	Humanized, PEGylated Fab	Autoimmune inflammatory diseases, including rheumatoid arthritis, Crohn’s disease, and psoriatic arthritis	Cimzia	Unknown, preliminary studies suggest lack of efficacy as well as risk factors	[120]
Golimumab	TNFα	Fully Human IgG1	Autoimmune inflammatory diseases, including rheumatoid arthritis, Crohn’s disease, and psoriatic arthritis	Simponi	Unknown, preliminary studies suggest lack of efficacy as well as risk factors	[121]

## Data Availability

Not applicable.
